# Supplementary material for: Protective Effect of Methyl Sulfonyl Methane on the Progression of Age-Induced Bone Loss by Regulating Oxidative Stress-Mediated Bone Resorption
Source: Antioxidants (Basel). 2025 Feb 13;14(2):216. doi: 10.3390/antiox14020216 (PMC11851857; doi:10.3390/antiox14020216)
Supplement: Supplementary file 1 [file antioxidants-14-00216-s001.zip › antioxidants-3429637-supplementary.pdf]

antioxidants

Article

Protective Effect of Methyl Sulfonyl Methane on the Progression of Age-Induced Bone Loss by Regulating Oxidative Stress Mediated Bone Resorption

Duo Zhang [1], Leilei Wang [2], Lu Tang [1], Yeting Zhang [1], Huaiyong Zhang [2,3,*] and Lin Zou [1,*]

[1] Physical education department, Civil aviation flight university of China, Guanghan 618307, China; summer@cafuc.edu.cn (D.Z.); lutangcafuc@163.com (L.T.); zhangyt@cafuc.edu.cn (Y.Z.)
[2] Key laboratory of animal biochemistry and nutrition of agriculture ministry, College of animal science and technology, Henan agricultural university, Zhengzhou 450046, China; wl911152@163.com (L.W.)
[3] Laboratory for animal nutrition and animal Product quality, Department of animal sciences and aquatic ecology, Ghent university, Ghent, 9000, Belgium
* Correspondence: huaiyong.zhang@ugent.be (H.Z.); zoulin@cafuc.edu.cn (L.Z.)

Table S1. Composition and calculated nutrient content (as fed basis)

Ingredients	Content, g/kg	Nutrient	Measured content, %
Corn starch	397.5	Total energy, kJ/g	15.07
Casein	200.0	Protein	17.90
Maltodextrin	132.0	Fat	7.00
Sucrose	100.0	Carbohydrate	64.40
Soybean oil	70.0	Calcium	0.51
Cellulose	50.0	Phosphorus	0.32
Mineral mix [a]	35.0		
Vitamin mix [b]	10.0		
L-cystine	3.0		
Choline chloride	2.5		
Total	1000.0		

[a] Mineral mix included: Magnesium, 0.05%; Iron, mg/kg; Zinc, 35 mg/kg; Manganese, 11 mg/kg; Copper, 6 mg/kg; Selenium, 0.24 mg/kg.

[b] Vitamin mix contained: Vitamin A, 4 IU/g; Vitamin D3, 1 IU/g; Vitamin E, 81.6 IU/kg; Vitamin K, 0.75 mg/kg; Thiamine, 4.8 mg/kg; Riboflavin, 6.7 mg/kg; Niacin, 30 mg/kg; Pantothenic acid, 16 mg/kg; Folic acid, 2.1 mg/kg; Pyridoxine, 5.8 mg/kg; Biotin,0.2 mg/kg; Vitamin B12, 28 mg/kg

Table S2. Primers for quantitative real-time PCR.

Genes	Gene ID	Primer	Sequence (5′-3′)	Size (bp)
V-ATPase	NM_175406.3	Reverse	tgagtttgaggccgacagac	131
		Forward	ctaacaaccgcaacccctct	
MMP9	NM_013599.5	Reverse	cgctcatgtacccgctgtat	136
		Forward	tgtggttcagttgtggtggt	
Ctsk	NM_007802.4	Reverse	tgtgtccatcgatgcaagct	112
		Forward	atagcccaccaccaacactg	
OPG	NM_001411506.1	Reverse	aaacacacgaactgcagcac	92
		Forward	gtggcttctctgtttccgga	
RANKL	NM_011613.4	Reverse	tcgggaagcgtacctacaga	102
		Forward	cgtgctccctcctttcatca	
NFATc1	NM_001164109.1	Reverse	gaccgaagatacctggctcg	96
		Forward	aggtccagagtgctatcggt	
Col1a1	NM_007742.4	Reverse	tagaggctctgaaggtcccc	143
		Forward	caccagcaataccaggagca	
Phex	NM_011077.2	Reverse	cagcgctatgattccccagt	106
		Forward	caccacattctccgagggac	
Sost	NM_024449.6	Reverse	acaaccagaccatgaaccgg	86
		Forward	gcagctgtactcggacacat	
Runx2	NM_001145920.3	Reverse	ccctgaactctgcaccaagt	147
		Forward	tggagtggatggatggggat	
Sp7	NM_001348205.1	Reverse	accagaagcgaccacttgag	119
		Forward	ttggcttcttcttccccgac	
Nrf2	NM_010902.5	Reverse	aacagaacggccctaaagca	142
		Forward	ccaggactcacgggaacttc	
HO-1	NM_010442.2	Reverse	agccccaccaagttcaaaca	141
		Forward	catcacctgcagctcctcaa	
SOD1	NM_011434.2	Reverse	agacctgggcaatgtgactg	146
		Forward	ttccacctttgcccaagtca	
SOD2	NM_013671.3	Reverse	gagtccaaggttcaggctgg	124
		Forward	atccccagcagcggaataag	
GPx1	NM_001329527.1	Reverse	cagtccaccgtgtatgcctt	123
		Forward	gatcgtggtgcctcagagag	
GPx3	NM_001329860.1	Reverse	gtgaacggggagaaagagca	130
		Forward	agttccagcggatgtcatgg	
CAT	NM_009804.2	Reverse	atggtcaccggcacatgaat	137
		Forward	ccttcctgcctctccaacag	
GR	NM_010344.4	Reverse	ccaccacgaccatgattcca	114
		Forward	ggcccttttcatccgtctga	
NF-κB	NM_001410442.1	Reverse	aggatttgctgagggttggg	136
		Forward	ataaggggcgctgcttttct	
IL-1β	NM_008361.4	Reverse	ccagcttcaaatctcgcagc	98
		Forward	aagacacaggtagctgccac	
IL-6	NM_001314054.1	Reverse	ttgggactgatgctggtgac	91
		Forward	caggtctgttgggagtggtat	
TNF-α	NM_001278601.1	Reverse	ggtgcctatgtctcagcctc	131
		Forward	actgatgagagggaggccat	

β-actin	NM_007393.5	Reverse	tcaacaccccagccatgtac	87
		Forward	cggagtccatcacaatgcct	
GAPDH	NM_001289726.2	Reverse	catcttccaggagcgagacc	145
		Forward	ggcggagatgatgacccttt	

V-ATPase, vacuolar-type H^+-ATPase; *MMP9*, matrix metallopeptidase 9; *Ctsk*, cathepsin K; *OPG*, osteoprotegerin; *RANKL*, receptor activator of nuclear factor-κ B ligand; *NFATc1*, nuclear factor of activated T-cells, cytoplasmic, calcineurin dependent 1; *Col1a1*, collagen type I alpha 1; Phex, phosphate regulating endopeptidase homolog x-linked; *Sost*, sclerostin; *Runx2*, runt related transcription factor 2; *Sp7*, Sp7 transcription factor 7; *Nrf2*, nuclear factor E2 related factor 2; *HO-1*, heme oxygenase 1; *SOD*, superoxide dismutase ; *GPx*, glutathione peroxidase; *CAT*, catalase; *GR*, glutathione reductase; *NF-κB*, nuclear factor-kappa B; *IL*, interleukin; *TNF-α*, tumor necrosis factor-alpha; *GAPDH*, glyceraldehyde-3-phosphate dehydrogenase.

Table S3. Correlations between bone characteristics and oxidative status

Oxidative status	Bone characteristics	r	p-value	r.sign
MDA	BV/TV	-0.6208	0.0060	Negative
MDA	Tb.N	-0.6780	0.0020	Negative
MDA	Tb.Sp	0.6711	0.0023	Positive
MDA	Tb.Th	-0.0958	0.7053	Negative
MDA	Tb.Cd	-0.6723	0.0022	Negative
SOD	BV/TV	0.6296	0.0051	Positive
SOD	Tb.N	0.4198	0.0829	Positive
SOD	Tb.Sp	-0.5860	0.0106	Negative
SOD	Tb.Th	0.4078	0.0930	Positive
SOD	Tb.Cd	0.5695	0.0136	Positive
GPx	BV/TV	0.4619	0.0537	Positive
GPx	Tb.N	0.5450	0.0193	Positive
GPx	Tb.Sp	-0.2673	0.2836	Negative
GPx	Tb.Th	0.2226	0.3747	Negative
GPx	Tb.Cd	0.4354	0.0709	Positive

Correlation analysis was performed using Pearson method, and statistical significance was defined as $p < 0.05$.

MDA, Malondialdehyde; SOD, Superoxide dismutase; GPx, Glutathione peroxidase; BV/TV, the ratio of bone volume to total volume; Tb.N, trabecular number; Tb.Sp, trabecular spaces; Tb.Th, trabecular thickness; Tb.Cd, trabecular connectivity density.

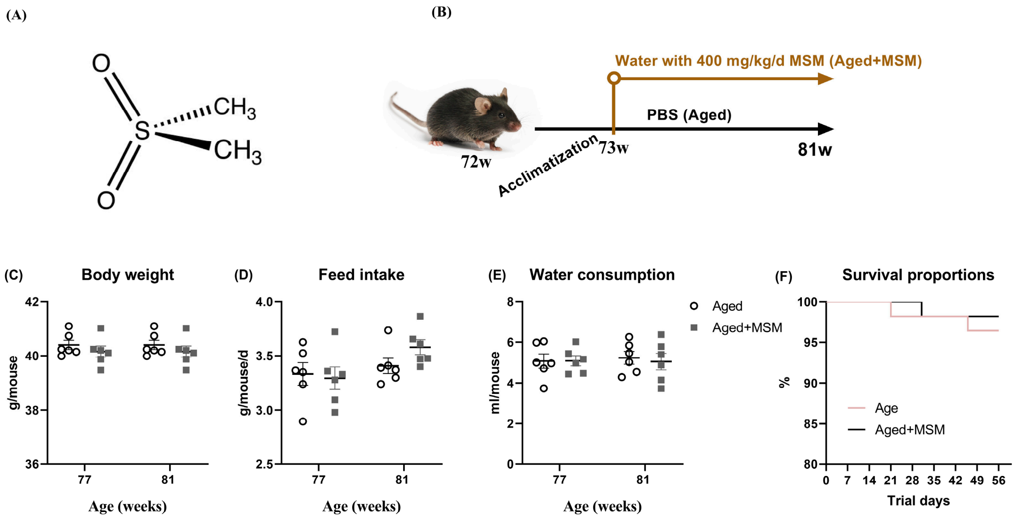

Figure S1. (A) Chemical structure of methyl sulfonyl methane (MSM) and **(B)** the experimental design used to explore the effects of MSM on the growth of mice. Effects of MSM on the growth of mice including **(C)** body weight, **(D)** feed intake, (**E)** water consumption, and (**F**) survival proportions during weeks 73 to 81. Survival analysis was performed using the Kaplan-Meier method. All the results were shown as mean ± standard deviation. Statistical significance was defined as $p < 0.05$.
